# Investigating the relationship between substance use and sexual behaviour in young people in Britain: findings from a national probability survey

**DOI:** 10.1136/bmjopen-2016-011961

**Published:** 2016-06-30

**Authors:** S N Khadr, K G Jones, S Mann, D R Hale, A M Johnson, R M Viner, C H Mercer, K Wellings

**Affiliations:** 1 Population, Policy and Practice Programme, University College London Institute of Child Health, London, UK; 2 Research Department of Infection and Population Health, University College London, London, UK; 3 Research Department of Reproductive Health, University College London Institute of Women's Health, London, UK; 4 Department of Social and Environmental Health Research, London School of Hygiene and Tropical Medicine, London, UK

**Keywords:** EPIDEMIOLOGY, GENITOURINARY MEDICINE, PUBLIC HEALTH

## Abstract

**Background:**

Health risk behaviours are prominent in late adolescence and young adulthood, yet UK population-level research examining the relationship between drug or alcohol use and sexual health and behaviour among young people is scarce, despite public health calls for an integrated approach to health improvement. Our objective was to further our understanding of the scale of and nature of any such relationship, using contemporary data from Britain's third National Survey of Sexual Attitudes and Lifestyles (Natsal-3).

**Methods:**

Analyses of data from Natsal-3, a stratified probability survey of 15 162 men and women (3869 aged 16–24 years), undertaken in 2010–2012, using computer-assisted personal interviewing, were carried out. Logistic regression was used to explore associations between reporting (1) frequent binge drinking (≥weekly), (2) recent drug use (within past 4 weeks) or (3) multiple (both types of) substance use, and key sexual risk behaviours and adverse sexual health outcomes. We then examined the sociodemographic profile, health behaviours and attitudes reported by ‘risky’ young people, defined as those reporting ≥1 type of substance use plus non-condom use at first sex with ≥1 new partner(s), last year.

**Results:**

Men and women reporting frequent binge drinking or recent drug use were more likely to report: unprotected first sex with ≥1 new partner(s), last year; first sex with their last partner after only recently meeting; emergency contraception use (last year) and sexually transmitted infection diagnosis/es (past 5 years). Associations with sexual risk were frequently stronger for those reporting multiple substance use, particularly among men. The profile of ‘risky’ young people differed from that of other 16–24 years old.

**Conclusions:**

In this nationally representative study, substance use was strongly associated with sexual risk and adverse sexual health outcomes among young people. Qualitative or event-level research is needed to examine the context and motivations behind these associations to inform joined-up interventions to address these inter-related behaviours.

**Strengths and limitations of this study**

- This is the first British study to show the strength and breadth of association between reported drug or alcohol use, and sexual health and behaviour, and the impact of multiple substance use, in a nationally representative sample of young people.
- Profiling risky young people allowed us to identify potential contextual and individual risk factors for the clustering of substance use and sexual risk behaviour.
- The third National Survey of Sexual Attitudes and Lifestyles (Natsal-3) was a cross-sectional survey, so causality cannot be assumed from any of the data.
- We were not able to explore the effects of frequency of drug use, nor were we able to consider the impact of different types of drugs on associations with sexual health and behaviour.
- Multivariable analyses were not performed to assess for confounding variables in this descriptive study, as we sought to identify risk factors to inform the targeting and tailoring of interventions, rather than to identify explanatory variables.

## Introduction

Britain ranks low among other wealthy European nations, regarding young people's sexual health.^1^ The UK's under-18 birth rate remains the highest in Western Europe,^2^ and those aged 16–24 years account for more than half of sexually transmitted infections (STIs) diagnosed in England annually.^3^

Most adults in the UK drink alcohol, and while young people drink less frequently than older adults, when they drink, they do so more heavily.^4^ Research shows that over 40% of young people who drink alcohol in a given week report binge drinking on at least one day.^4^ Levels of illicit drug use are also highest among this age group.^5^ Peak ages of onset for cannabis, cocaine and ecstasy use are between 16 and 18 years, with the average age of desistance between 24 and 26 years.^5^

While many studies have explored associations between substance use and risky sexual behaviours during adolescence, few of any scale have been conducted in Britain. The second National Survey of Sexual Attitudes and Lifestyles (Natsal-2), undertaken between 1999 and 2001, among those aged 16–44 years and resident in Britain, observed higher partner numbers and more unsafe sex among heavy drinkers.^6^ However, the applicability of these findings to young people today is unknown.

The potential benefits of adopting an integrated approach to health improvement, addressing related behaviours such as substance use in tandem with sexual behaviours, are now recognised in UK sexual health policy.^7^ Research is needed to improve understanding of what shapes sexual risk-taking behaviour, particularly the role of alcohol and drugs, and to support the development of effective interventions. In this study, we examine the relationship between substance use, sexual risk behaviours and adverse sexual health outcomes among young people in Britain, using contemporary data from Natsal-3.

## Methods

### Study design

Natsal-3 is a stratified probability sample survey of 15 162 men and women aged 16–74 years (3869 participants aged 16–24 years), resident in Britain, undertaken between September 2010 and August 2012. Details of the sampling methodology and data collection are published elsewhere.^8^ ^9^ Briefly, participants were interviewed using a combination of face-to-face computer-assisted personal interviewing and computer-assisted self-interview (CASI), with the more sensitive questions asked in the CASI.^8^ The response rate was 57.7%,^8^ in line with other major social surveys completed in Britain around the same time.^10^ ^11^ The cooperation rate (number of interviews completed from eligible addresses for which contact was made) was 65.8%.^8^ Differences in response by area deprivation were assessed using the Index of Multiple Deprivation (IMD).^12^ Response rates showed little variation by IMD quintile.^8^

### Sample

Participants aged 16–24 years at interview were the denominator for these analyses, hereafter referred to as ‘young people’, ‘young men’ or ‘young women’, as appropriate.

### Measures

Two measures of recent substance use were considered: (1) frequent binge drinking (defined as at least once a week) based on current drinking practices and (2) recent non-prescribed drug use (in the past 4 weeks). An episode of binge drinking was defined as consumption of more than six (for women) or eight (for men) units of alcohol on any one occasion.^13^ Drugs asked about included cannabis, amphetamines, cocaine, crack cocaine, ecstasy, heroin, lysergic acid diethylamide (LSD), crystal methamphetamine and amyl nitrates. A measure of multiple substance use was created by combining frequent binge drinking and recent drug use.

We included the following measures of sexual risk behaviour and adverse sexual health outcomes for which Natsal-3 collected data:

1. Non-use of condoms at first sex with one or more new partners in the last year;
2. First sex with the most recent partner having occurred after they had ‘only just’ or ‘just recently’ met;
3. Emergency contraception use with a partner in the last year;
4. Any STI diagnosis/es in the past 5 years;
5. Ever-experience of abortion;
6. Ever-experience of attempted non-volitional sex.

Last, a composite measure of sexual risk and substance use was created. Young people reporting (1) frequent binge drinking or recent drug use (or both), as well as (2) non-use of condoms at first sex with one or more new partners in the last year, were described as ‘risky young people’. In the absence of a single ‘best’ measure of sexual risk, non-condom use among young people during ‘higher risk’ sex (with a non-cohabiting partner) is a key indicator of sexual risk behaviour.^14^

The profile of risky young people was compared with that of other young people in terms of the following key demographic variables: ethnic group; religiosity; educational level; family structure at 14 years of age and area-level deprivation score, using the IMD, a multidimensional measure combining income, employment, health, education, access to housing and services, and crime and living environments.^12^ Health variables included smoking status and depression, the latter assessed using the Patient Health Questionnaire-2 (PHQ-2).^15^ Contextual measures included questions about where participants met their sexual partners and attitudinal measures eliciting opinions on pressure to have sex, sex without love, one-night stands and personal risk of STIs.

### Statistical analysis

Unadjusted bivariable logistic regression models were used to explore associations between each of frequent binge drinking, recent drug use or multiple substance use, and the aforementioned sexual risk behaviour and adverse sexual health outcome measures. The sociodemographic profile, health behaviours and attitudes of risky young people were then explored and compared with the remaining population of young people, using unadjusted bivariable logistic regression models. Multivariable analyses were not performed. The aims of this study were to describe the relationship between substance use and sexual health and outcomes, and to examine the profile of risky young people and identify potential risk factors, rather than to seek to identify explanatory variables.

All analyses used Stata V.12.1 (StataCorp. Stata Statistical Software: Release 12. College Station, Texas: StataCorp LP, 2011) and accounted for the probability weighting, clustering and stratification within the Natsal-3 sample. The data were weighted to correct for unequal selection probabilities and to broadly match the demographic profile of the British population figures, in terms of gender, age and Government Office Region, according to the 2011 UK Census.^8^

Data in this descriptive study are presented as percentages or crude (unadjusted) ORs with 95% CIs unless otherwise stated. Statistical significance is considered as p<0.05.

Participants provided oral informed consent for interviews.

## Results

The majority of young men (85.1%) and women (78.5%) interviewed currently drank alcohol (table 1), with 22.7% and 12.8% of young men and women, respectively, reporting binge drinking at least once a week. One in 5 young men and nearly 1 in 10 young women had used drugs in the past 4 weeks, by their report.

**Table 1:** Reported prevalence of substance use, sexual behaviours and adverse sexual health outcomes among young people (16–24 years), by gender

	Young men			Young women		
	Per cent	95% CI	Denominator*†‡	Per cent	95% CI	Denominator*†‡
*Substance use*						
Drink alcohol currently	85.1	(82.8 to 87.1)	1729, 1238	78.5	(76.3 to 80.5)	2140, 1207
Binge drink at least weekly	22.7	(20.5 to 25.1)	1727, 1236	12.8	(11.3 to 14.6)	2138, 1206
Drug use in past 4 weeks§	20.2	(18.1 to 22.6)	1607, 1155	9.6	(8.3 to 11.2)	2001, 1119
*Sexual experience and behaviours*						
Ever had sex	80.7	(78.4 to 82.7)	1686, 1212	80.6	(78.5 to 82.5)	2072, 1167
1+ new partner in last year	47.3	(44.4 to 50.1)	1695, 1214	38.9	(36.6 to 41.4)	2102, 1185
No condom used at first sex with 1+ new partner(s), last year	23.4	(21.3 to 25.7)	1689, 1210	23.0	(21.1 to 25.1)	2097, 1182
Just/recently met last partner at first sex¶	29.1	(26.4 to 31.9)	1371, 999	21.8	(19.6 to 24.0)	1730, 963
*Adverse sexual health outcomes*						
EC** use with a partner, last year	5.8	(4.7 to 7.2)	1712, 1227	5.4	(4.3 to 6.8)	2091, 1180
STI diagnosis††, past 5 years	5.6	(4.5 to 7.0)	1703, 1216	10.9	(9.45 to 12.6)	2107, 1189
Ever had an abortion	–	–	–	7.9	(6.85 to 9.2)	2124, 1198
Ever experienced attempted non-volitional sex	3.7	(2.85 to 4.9)	1688, 1208	16.4	(14.75 to 18.3)	2078, 1172

*The denominator for all variables is all 16–24 years old (by gender) unless otherwise specified.

†Unweighted, weighted denominators.

‡Denominators may vary due to missing data/non-response.

§The denominator for this variable is all 16–24 years old who report ever having had some sexual experience, by gender.

¶The denominator for this variable is all 16–24 years old who report ever having had heterosexual sex, by gender.

**Emergency contraception (EC).

††Excludes thrush.

STI, sexually transmitted infection.

Four in five young people had ever had sex with someone of the same or opposite sex, with 47% of young men and 38.9% of young women reporting at least one new sexual partner in the last year. Similar proportions of young men (23.4%) and women (23%) reported non-condom use at first sex with at least one new partner in the last year. The prevalence of adverse sexual health outcomes varied by gender, with young women nearly twice as likely to report an STI diagnosis within the past 5 years as men. One in six young women gave a history of previous experience of attempted non-volitional sex, compared with 3.7% of young men.

### Associations between substance use behaviours and sexual risk behaviours or adverse sexual health outcomes

There were strong associations between reporting any one type of substance use behaviour and higher levels of sexual risk, across both genders (table 2). Participants reporting frequent binge drinking or recent drug use were more likely to report non-condom use at first sex with one or more new partners in the last year and having first sex with their last partner after they had only recently met. There were also strong associations with reporting adverse sexual health outcomes including emergency contraception use with a partner within the last year and a STI diagnosis in the past 5 years, for both genders. Young women reporting recent drug use were significantly more likely to report previous experience of abortion and/or attempted non-volitional sex than women who reported frequent binge drinking or neither behaviour.

**Table 2:** Reported prevalence of sexual risk behaviours and adverse sexual health outcomes in relation to substance use behaviour among young people, by gender

	Of those reporting				
Per cent (95% CI) to report	No substance use behaviour	Binge drinking (weekly), only	Drug use (past 4 weeks),* only	Multiple (both) behaviours*	p Value
*Men*					
No condom used at first sex with 1+ new partner(s), last year					<0.0001
Per cent (95% CI)	18.5% (15.9 to 21.4)	31.2% (25.1 to 37.9)	35.1% (27.9 to 43.1)	47.9% (38.8 to 57.1)	
OR† (95% CI)	1.00	2.00 (1.38 to 2.88)	2.39 (1.64 to 3.48)	4.05 (2.71 to 6.05)	
*Denominators*‡§	*1014, 725*	*244, 180*	*193, 136*	*128, 94*	
Just/recently met last partner at first sex					<0.0001
Per cent (95% CI)	22.9% (19.7 to 26.5)	39.9% (33.3 to 46.8)	34.7% (27.7 to 42.4)	39.2% (30.2 to 48.9)	
OR (95% CI)	1.00	2.23 (1.59 to 3.12)	1.79 (1.22 to 2.62)	2.17 (1.40 to 3.35)	
*Denominators*	*811, 594*	*240, 178*	*189, 133*	*128, 94*	
EC¶ use with a partner, last year					<0.0001
Per cent (95% CI)	3.6% (2.6 to 4.9)	10.1% (6.6 to 15.1)	9.8% (5.6 to 16.8)	13.9% (8.7 to 21.6)	
OR (95% CI)	1.00	3.00 (1.69 to 5.31)	2.91 (1.47 to 5.73)	4.31 (2.34 to 7.97)	
*Denominators*	*1021, 731*	*245, 181*	*195, 137*	*129, 95*	
STI diagnosis,** past 5 years					<0.0001
Per cent (95% CI)	3.5% (2.5 to 5.0)	9.0% (5.7 to 13.9)	9.9% (5.8 to 16.6)	13.5% (8.0 to 21.9)	
OR (95% CI)	1.00	2.68 (1.46 to 4.92)	3.00 (1.51 to 5.96)	4.24 (2.17 to 8.28)	
*Denominators*	*1025, 733*	*244, 180*	*195, 134*	*129, 95*	
Ever experienced attempted non-volitional sex					0.1499
Per cent (95% CI)	3.6% (2.6 to 5.1)	2.3% (0.9 to 5.4)	7.5% (4.1 to 13.5)	4.3% (1.6 to 11.1)	
OR (95% CI)	1.00	0.62 (0.24 to 1.58)	2.15 (1.02 to 4.51)	1.20 (0.41 to 3.48)	
*Denominators*	*1019, 730*	*242, 179*	*194, 134*	*127, 92*	
*Women*					
No condom used at first sex with 1+ new partner(s), last year					<0.0001
Per cent (95% CI)	21.1% (18.9 to 23.3)	41.0% (33.6 to 48.8)	30.0% (22.4 to 39.0)	47.6% (34.3 to 61.4)	
OR† (95% CI)	1.00	2.60 (1.87 to 3.63)	1.61 (1.06 to 2.44)	3.41 (1.96 to 5.93)	
*Denominators*‡§	*1563, 878*	*210, 117*	*130, 72*	*66, 35*	
Just/recently met last partner at first sex					<0.0001
Per cent (95% CI)	18.0% (15.7 to 20.5)	30.9% (24.6 to 38.0)	34.6% (25.6 to 44.8)	45.5% (32.3 to 59.3)	
OR (95% CI)	1.00	2.04 (1.43 to 2.91)	2.42 (1.53 to 3.82)	3.81 (2.17 to 6.69)	
*Denominators*	*1322, 740*	*209, 117*	*128, 71*	*65, 34*	
EC¶ use with a partner, last year					0.0199
Per cent (95% CI)	4.9% (3.8 to 6.4)	9.1% (5.3 to 15.2)	9.1% (4.7 to 16.6)	12.0% (5.5 to 24.3)	
OR (95% CI)	1.00	1.94 (1.05 to 3.56)	1.92 (0.92 to 4.00)	2.63 (1.09 to 6.32)	
*Denominators*	*1545, 868*	*211, 118*	*129, 71*	*66, 35*	
STI diagnosis,** past 5 years					<0.0001
Per cent (95% CI)	9.2% (7.6 to 11.0)	18.9% (13.8 to 25.4)	24.8% (16.9 to 34.9)	21.7% (12.6 to 34.9)	
OR (95% CI)	1.00	2.32 (1.52 to 3.53)	3.27 (1.94 to 5.51)	2.75 (1.43 to 5.30)	
*Denominators*	*1573, 884*	*211, 118*	*131, 72*	*66, 35*	
Ever had an abortion					<0.0001
Per cent (95% CI)	6.9% (5.7 to 8.4)	9.6% (6.0 to 14.9)	20.8% (13.9 to 30.0)	17.7% (9.7 to 30.2)	
OR (95% CI)	1.00	1.42 (0.83 to 2.42)	3.53 (2.11 to 5.90)	2.90 (1.44 to 5.83)	
*Denominators*	*1588, 892*	*210, 118*	*132, 73*	*66, 35*	
Ever experienced attempted non-volitional sex					<0.0001
Per cent (95% CI)	15.1% (13.2 to 17.2)	20.1% (14.4 to 27.3)	38.7% (29.5 to 48.8)	29.6% (19.4 to 42.8)	
OR (95% CI)	1.00	1.42 (0.92 to 2.17)	3.55 (2.28 to 5.51)	2.36 (1.33 to 4.18)	
*Denominators*	*1556, 875*	*205, 115*	*126, 69*	*64, 34*	

The denominator for this variable is all 16-24 year olds who report ever having had heterosexual sex, by gender.

†ORs are crude (unadjusted) ORs.

‡Unweighted, weighted denominators.

§Denominators may vary due to missing data/non-response.

¶Emergency contraception (EC).

**Excludes thrush.

STI, sexually transmitted infection.

Associations with sexual risk were frequently stronger in those reporting multiple substance use, particularly among men. Most notable across both genders was the increased likelihood of reporting non-condom use at first sex with a new partner in the last year, with ORs of 4.05 and 3.41 in young men and women reporting multiple substance use relative to those reporting neither type of use. Similar findings were observed for young women reporting first sex with their last partner after they had only recently met (OR 3.81). Those reporting multiple substance use were more likely to have used emergency contraception with a partner in the last year (ORs of 4.31 in men and 2.63 in women), and young men were more likely to report one or more STI diagnoses in the past 5 years (OR 4.24).

### Characteristics of young people reporting risky sexual behaviour and substance use

Tables 3 and 4 describe the characteristics of risky young men and women, respectively, defined here as those reporting one or more types of substance use and non-use of condoms at first sex with at least one new partner in the last year, compared with other 16–24 years old. The data show that risky young people were more likely to be white, and less religious. There was no association with area-level deprivation score, but they were more likely to have left school at the age of 16 years. Risky young men and women were more likely to have met their most recent partner in a pub, bar, club or overseas. They also were more likely to report having had a new sexual partner from overseas while outside the UK, and the young men were more likely to have paid for sex while overseas. Men and women in the ‘risky’ group were more likely to be smokers and the young women were more likely to report current symptoms of depression. They were less likely to disapprove of one-night stands and sex without love, and more likely to consider themselves at risk of acquiring an STI. They were more likely to have had their sexual debut before 16 years of age.

**Table 3:** Characteristics of risky young men* relative to other young men

	All other young men		Risky young men*†				
*Unweighted, weighted denominators*	*1515, 1089*		*214, 149*				
Mean (SD) age at interview	19.9 (2.5)		20.3 (2.2)				
	Per cent	95% CI	Per cent	95% CI	OR‡	95% CI	p Value
*Sociodemographic profile*							
Ethnic group							0.0207
White	83.1	(80.5 to 85.5)	93.3	(87.0 to 96.7)	1.00		
Black	3.5	(2.6 to 4.8)	2.4	(0.7 to 7.8)	0.61	(0.18 to 2.13)	
Other	13.3	(11.2 to 15.8)	4.3	(1.8 to 10.0)	0.29	(0.12 to 0.71)	
Religion important and attendance regular							0.0030
No	90.4	(88.4 to 92.1)	98.6	(94.8 to 99.6)	1.00		
Yes	9.6	(7.9 to 11.6)	1.4	(0.4 to 5.2)	0.14	(0.04 to 0.51)	
Index of Multiple Deprivation quintiles^12^							0.1599
1–2 (least deprived)	37.4	(34.4 to 40.5)	30.2	(23.9 to 37.4)	1.00		
3	18.0	(15.9 to 20.3)	20.9	(15.8 to 27.3)	1.44	(0.94 to 2.20)	
4–5 (most deprived)	44.6	(41.3 to 47.8)	48.8	(41.5 to 56.3)	1.36	(0.95 to 1.94)	
Educational attainment§							<0.0001
Left school aged 17+ years	79.4	(76.6 to 81.9)	62.9	(54.8 to 70.2)	1.00		
Left at age 16 years; some qualifications	16.5	(14.2 to 19.1)	28.1	(21.5 to 35.8)	2.15	(1.44 to 3.19)	
Left at age 16 years; no qualifications	4.1	(3.1 to 5.5)	9.0	(5.5 to 14.5)	2.77	(1.49 to 5.15)	
Lived with both natural parents to age 14 years							0.0414
Yes	72.4	(69.9 to 74.7)	65.4	(58.5 to 71.8)	1.00		
No	27.6	(25.3 to 30.1)	34.6	(28.2 to 41.5)	1.38	(1.01 to 1.89)	
*Sexual behaviour*							
Had sex before age 16 years							<0.0001
No	71.5	(68.8 to 74.0)	49.2	(41.6 to 56.7)	1.00		
Yes	28.5	(26.0 to 31.2)	50.8	(43.3 to 58.4)	2.59	(1.87 to 3.58)	
Where met most recent partner							0.0002
Other	87.0	(84.7 to 89.0)	76.1	(69.4 to 81.8)	1.00		
Pub/bar/club/on holiday	13.0	(11.0 to 15.3)	23.9	(18.2 to 30.6)	2.10	(1.42 to 3.11)	
Foreign sex partners abroad, past 5 years							<0.0001
No	88.6	(86.7 to 90.2)	69.8	(62.2 to 76.5)	1.00		
Yes	11.4	(9.8 to 13.3)	30.2	(23.5 to 37.8)	3.34	(2.30 to 4.87)	
Ever paid for sex outside the UK							<0.0001
No	98.5	(97.6 to 99.0)	91.4	(85.3 to 95.0)	1.00		
Yes	1.5	(1.0 to 2.4)	8.6	(5.0 to 14.7)	6.16	(2.90 to 13.07)	
*Health*							
Smoking status							<0.0001
Never smoked	62.8	(59.9 to 65.7)	30.3	(23.8 to 37.5)	1.00		
Ex-smoker	8.7	(7.2 to 10.4)	10.7	(6.7 to 16.6)	2.55	(1.43 to 4.55)	
Current smoker	28.5	(25.8 to 31.3)	59.1	(51.4 to 66.4)	4.31	(3.01 to 6.17)	
Current depression (PHQ-2)^15^							0.4660
No	89.4	(87.5 to 91.1)	87.5	(81.7 to 91.7)	1.00		
Yes	10.6	(8.9 to 12.5)	12.5	(8.3 to 18.3)	1.20	(0.73 to 1.98)	
	Per cent	95% CI	Per cent	95% CI	OR‡	95% CI	p Value
*Attitudes*							
People are under pressure to have sex							0.6158
No	31.7	(29.2 to 34.4)	33.6	(27.1 to 40.8)	1.00		
Yes	68.3	(65.6 to 70.8)	66.4	(59.2 to 72.9)	0.92	(0.66 to 1.28)	
Sex without love OK							<0.0001
No	38.8	(36.2 to 41.5)	13.3	(9.3 to 18.8)	1.00		
Yes	61.2	(58.5 to 63.8)	86.7	(81.2 to 90.7)	4.13	(2.70 to 6.32)	
One-night stands OK							<0.0001
No	68.8	(66.1 to 71.5)	46.6	(39.2 to 54.1)	1.00		
Yes	31.2	(28.5 to 33.9)	53.4	(45.9 to 60.8)	2.54	(1.83 to 3.51)	
At personal STI risk							<0.0001
No	92.1	(90.3 to 93.6)	71.3	(64.0 to 77.7)	1.00		
Yes	7.9	(6.4 to 9.7)	28.7	(22.3 to 36.0)	4.70	(3.16 to 6.98)	

*Young men reporting (1) binge drinking at least once a week, drug use in the past 4 weeks, or both, as well as (2) non-condom use at first sex with 1+ new partner(s) in the last year.

†The denominator for the drug use variable informing this composite measure is all 16–24-year-old men reporting ever having had some sexual experience.

‡ORs are crude (unadjusted) ORs.

§The denominator for this variable is all those aged ≥17 years.

PHQ-2, Patient Health Questionnaire-2; STI, sexually transmitted infection.

**Table 4:** Characteristics of risky young women* relative to other young women

	All other young women		Risky young women*†				
*Unweighted, weighted denominators*	*1971, 1120*		*169, 86*				
Mean (SD) age at interview	20.1 (2.6)		20.0 (2.3)				
	Per cent	95% CI	Per cent	95% CI	OR‡	95% CI	p Value
*Sociodemographics*							
Ethnic group							0.0019
White	81.4	(79.0 to 83.6)	93.7	(88.8 to 96.6)	1.00		
Black	5.0	(4.0 to 6.3)	0.6	(0.1 to 4.4)	0.11	(0.01 to 0.79)	
Other	13.5	(11.5 to 15.9)	5.6	(3.0 to 10.5)	0.36	(0.18 to 0.72)	
Religion important and attendance regular							0.0243
No	90.8	(88.9 to 92.4)	96.8	(92.0 to 98.8)	1.00		
Yes	9.2	(7.6 to 11.1)	3.2	(1.2 to 8.0)	0.32	(0.12 to 0.86)	
Index of Multiple Deprivation quintiles^12^							0.7809
1–2 (least deprived)	34.0	(31.5 to 36.6)	34.4	(26.3 to 43.5)	1.00		
3	20.6	(18.3 to 23.1)	18.1	(12.4 to 25.7)	0.87	(0.52 to 1.45)	
4–5 (most deprived)	45.4	(42.7 to 48.1)	47.4	(38.6 to 56.5)	1.03	(0.68 to 1.57)	
Educational attainment§							0.0400
Left school aged 17+ years	80.6	(78.5 to 82.5)	73.7	(65.9 to 80.3)	1.00		
Left at age 16 years; some qualifications	15.1	(13.3 to 17.0)	17.8	(12.1 to 25.4)	1.29	(0.81 to 2.06)	
Left at age 16 years; no qualifications	4.4	(3.5 to 5.5)	8.5	(4.9 to 14.3)	2.14	(1.14 to 4.02)	
Lived with both natural parents to age 14 years							0.0898
Yes	65.8	(63.5 to 68.1)	59.0	(51.1 to 66.4)	1.00		
No	34.2	(31.9 to 36.5)	41.0	(33.6 to 48.9)	1.34	(0.96 to 1.87)	
*Sexual behaviour*							
Had sex before age 16 years							<0.0001
No	72.8	(70.5 to 75.0)	39.8	(31.8 to 48.3)	1.00		
Yes	27.2	(25.0 to 29.5)	60.2	(51.7 to 68.2)	4.05	(2.83 to 5.79)	
Where met most recent partner							0.0027
Other	86.8	(84.8 to 88.5)	77.8	(70.3 to 83.8)	1.00		
Pub/bar/club/on holiday	13.2	(11.5 to 15.2)	22.2	(16.2 to 29.7)	1.88	(1.25 to 2.83)	
Foreign sex partners abroad, past 5 years							0.0002
No	90.9	(89.2 to 92.3)	80.0	(71.7 to 86.4)	1.00		
Yes	9.1	(7.7 to 10.8)	20.0	(13.6 to 28.3)	2.49	(1.54 to 4.01)	
*Health*							
Smoking status							<0.0001
Never smoked	64.0	(61.6 to 66.3)	28.4	(21.2 to 37.0)	1.00		
Ex-smoker	9.0	(7.7 to 10.4)	7.8	(4.4 to 13.4)	1.95	(0.97 to 3.91)	
Current smoker	27.0	(24.9 to 29.3)	63.8	(55.2 to 71.7)	5.32	(3.55 to 7.99)	
Current depression (PHQ-2)^15^							0.0005
No	86.7	(85.0 to 88.3)	75.8	(67.9 to 82.3)	1.00		
Yes	13.3	(11.7 to 15.0)	24.2	(17.7 to 32.1)	2.08	(1.38 to 3.15)	
	Per cent	95% CI	Per cent	95% CI	OR‡	95% CI	p Value
*Attitudes*							
People are under pressure to have sex							0.4790
No	25.3	(23.1 to 27.7)	28.0	(21.3 to 35.8)	1.00		
Yes	74.7	(72.3 to 76.9)	72.0	(64.2 to 78.7)	0.87	(0.60 to 1.27)	
Sex without love OK							<0.0001
No	55.7	(53.1 to 58.2)	28.4	(21.5 to 36.6)	1.00		
Yes	44.3	(41.8 to 46.9)	71.6	(63.4 to 78.5)	3.17	(2.16 to 4.63)	
One-night stands OK							<0.0001
No	84.5	(82.5 to 86.2)	67.2	(59.0 to 74.5)	1.00		
Yes	15.5	(13.8 to 17.5)	32.8	(25.5 to 41.0)	2.65	(1.82 to 3.86)	
At personal STI risk							<0.0001
No	94.0	(92.6 to 95.2)	79.3	(70.6 to 86.0)	1.00		
Yes	6.0	(4.8 to 7.4)	20.7	(14.0 to 29.4)	4.12	(2.50 to 6.79)	

*Young women reporting (1) binge drinking at least once a week, drug use in the past 4 weeks, or both, as well as (2) non-condom use at first sex with 1+ new partner(s) in the last year.

†The denominator for the drug use variable informing this composite measure is all 16–24-year-old women reporting ever having had some sexual experience.

‡ORs are crude (unadjusted) ORs.

§The denominator for this variable is all those aged ≥17 years.

PHQ-2, Patient Health Questionnaire-2; STI, sexually transmitted infection.

## Discussion

### Principal findings

This is the first British study to examine links between both, drug and alcohol use, and sexual health and behaviour, in a nationally representative sample of young people. We observed strong associations between reporting frequent binge drinking or recent drug use and both, risky sexual behaviour and adverse sexual health outcomes. The strength of these associations was greater in young people reporting multiple substance use. Profiling of men and women engaging in risky sexual behaviour and substance use highlighted several areas for further exploration and potential future interventions.

### Strengths and limitations

Natsal-3 was a cross-sectional survey, so causality cannot be assumed from any of the data. However, as a large probability sample survey, its results can be considered to be broadly representative of the British general population. Response rates were comparable with other major social surveys in Britain around the same time, and item non-response was low, typically <5%, and usually 1–3%.^9^ Use of CASI is likely to have improved the quality of responses to sensitive questions on sexual behaviour and drug use. Showcards were used for questions on alcohol use and the circumstances of first sexual intercourse, so that participants only had to give a coded response, as per previous Natsal surveys.

Natsal-3 included more precise questions on alcohol use relative to previous Natsal surveys, and standardised drug use measures.^5^ Levels of alcohol and drug use reported by 16–24 years old in Natsal-3 were comparable with data for young people from other national surveys,^4^ ^5^ although fewer women completing Natsal-3 reported binge drinking at least once a week.^4^ Of note, only those reporting *some sexual experience* (∼93% of 16–24 years old surveyed) were invited to complete the CASI, in which the questions regarding drug use were asked. Hence, our drug use estimates might be slightly different than if we had asked *all* young people surveyed about their drug use, regardless of sexual experience.

We were not able to explore the effect of frequency of drug use on associations with sexual health and behaviour, and we did not examine associations by drug type.

The sexual behaviour variable chosen to inform the composite measure of substance use and sexual risk defining risky young people was non-condom use at first sex with one or more new partners in the last year. Non-condom use among young people during sex with a non-cohabiting partner is a key indicator of sexual risk behaviour,^14^ as in this situation, protection against STIs is usually advisable whether or not other contraception is in use. However, asking about condom use in one context does not necessarily capture correct use or consistency of condom use with casual partners, neither does it account for other dimensions of sexual behaviour that inform an individuals' risk profile.^14^ High partner numbers is another oft-used indicator of sexual risk, although frequency reports may be more susceptible to recall bias, or to gender-related reporting bias.^14^ ^16^

Multivariable analyses were not performed to assess for confounding variables in this descriptive study as we sought to identify risk factors to inform the targeting and tailoring of interventions, rather than to identify explanatory variables. Our aim was not to identify causal mechanisms, but to identify the extent to which risky behaviours cluster in a population, and to identify demographic and behavioural variables associated with that clustering, for further exploration in related studies.

### Comparisons with other studies of substance use and sexual health or behaviours

We observed strong relationships between substance use and both, sexual risk behaviours and adverse sexual health outcomes, although causality cannot be established. International cross-sectional studies have shown associations between substance use and unplanned sex or multiple partners;^17–20^ however, links with condom non-use are less clear^17^ ^18^ and may vary according to the type of substance used.^21^ ^22^

Data from event-level studies suggest that alcohol or cannabis use may not be directly responsible for non-condom use.^17^ ^23^ ^24^ Nevertheless, substance use is known to reduce inhibitions and impair judgement. UK research has highlighted geographical associations between alcohol-related hospital admissions and both, STI and teenage pregnancy rates,^25^ but longitudinal studies exploring the direction(s) of influence are lacking. International serial surveys suggest that substance use may predispose to, or develop subsequent to, an adverse sexual health outcome, as in the example of sexual violence.^26–29^

### Significance of multiple substance use

Young people engaging in multiple substance use were more likely to report risky sexual behaviours and adverse sexual health outcomes, particularly young men, although numbers were small. Models for the clustering of risk behaviours in young people include the so-called ‘gateway’ theory and a multiple risk behaviour ‘syndrome’ with common underlying psychosocial and developmental risk factors.^30^ Multiple substance use is likely to reflect a general propensity for risk-taking behaviour, potentially related to self-regulation capacity^31^ and influenced by brain development across adolescence.^32^ Our results are congruent with past studies demonstrating that concurrent multiple substance use is associated with higher novelty seeking, excessive substance use, addiction and injury.^33^ ^34^

### Characteristics of ‘risky’ young people

Profiling of risky young people identified several potential risk factors for the clustering of substance use and sexual risk behaviour, although these findings should be interpreted with caution, as multivariable analyses were not performed and numbers were small. White ethnicity appeared to be a risk factor, contrasting with findings from UK studies exploring associations between ethnicity and sexual behaviour only.^35^ ^36^ There was no association between risky phenotype and area-level deprivation, which is consistent with other Natsal-3 data^9^ and with national data on binge drinking patterns.^4^

We observed greater tendencies among risky young people to meet partners in bars, pubs and clubs or overseas, or to have paid for sex abroad. National survey data show that levels of drug use increase with frequency of nightclub and pub visits.^5^ Researchers have described the strategic use of drugs and alcohol for sex among young clubbers,^37^ ^38^ and observed associations with risky practices such as paying for sex^37^ and non-condom use.^39^

Risky young men and women were less likely to disapprove of one-night stands and sex without love, and more likely to consider themselves at risk of acquiring an STI. This may reflect peer group norms, impulsive or sensation-seeking personality traits, or modification of attitudes to be congruous with behaviour. Certain variables that might explain the clustering of risk behaviours were not measured in Natsal-3, limiting the scope for in-depth exploration of the impact of psychological or developmental factors on young people's health risk behaviours. Risky young women were more likely to report depressive symptoms, in what is likely to be a bi-directional relationship, although Hallfors *et al*^40^ found no evidence for depression-predicting substance use and risky sex.

### Implications for policy and further research

Despite greater recognition of their inter-relatedness,^7^ ^13^ joined-up policy initiatives targeting substance use and sexual behaviour remain limited. Potential implementation strategies include the routine provision of substance use advice in young people's sexual health services, in sex education, in university ‘freshers’ week’ packs and in online sexual health messaging. The strong associations between risk behaviours and social venues urge an intervention focus extending beyond individual risk behaviours to their broader social-structural influences, suggesting the need, for example, for universal provision of condom dispensers in bars, pubs and clubs.

Longitudinal or event-level studies are required to establish causality and directions of influence in the complex relationship between substance use and sexual risk. Qualitative studies in young people are relatively rare,^38^ ^41^ ^42^ particularly involving drug use. Further in-depth research is needed into the influence of social setting, cultural or socioeconomic factors, and psychological vulnerability, cognitive factors or personality traits on adolescent substance use and sexual behaviours, to further inform policy and interventions.

## Conclusion

In summary, substance use and sexual risk-taking are frequently related in young people, with strong associations between substance use and adverse sexual health outcomes. Despite good evidence of these links, contextual and motivational mechanisms behind associations between substance use and sexual behaviours in adolescence are poorly understood. Qualitative or event-level work is needed to examine the meaning of risk behaviours for young people themselves.
